# Assessing the ability of silage lactic acid bacteria to incorporate and transform inorganic selenium within laboratory scale silos

**DOI:** 10.1016/j.anifeedsci.2019.05.011

**Published:** 2019-06

**Authors:** Michael R.F. Lee, Hannah R. Fleming, Tristan Cogan, Chris Hodgson, David R. Davies

**Affiliations:** a Rothamsted Research, North Wyke, Okehampton, Devon, EX20 2SB, United Kingdom; b University of Bristol, Bristol Veterinary School, Langford, Somerset, BS40 5DU, United Kingdom; c Silage Solutions Ltd, Bwlch y Blaen, Ponthrydygroes, Ceredigion, SY25 6DP, United Kingdom

**Keywords:** CFU, colony forming units, DM, dry matter, FW, fresh weight, LAB, lactic acid bacteria, MRS, de man, rogosa and sharpe agar, NIRS, near infra-red spectroscopy, Nano-Se, elemental selenium, SeCys, selenocysteine, SeMet, selenomethionine, SeIV, selenite, SeVI, selenate, VFA, volatile fatty acid, Lactic acid bacteria, Silage inoculant, Nano-selenium, Inorganic selenium, Organic selenium, Silage quality

## Abstract

Selenium (Se) is a non-metallic trace element essential for normal cellular function, which has been linked with reduced risk of cancer, cardiovascular disease, cognitive decline and thyroid disease in humans. Se deficiency in livestock is associated with white muscle disease, retained placenta, ill-thrift and mastitis. Where Se status or bioavailability from the soil for plants is poor, livestock rely on supplemental Se in their diets predominantly as either sodium selenite (inorganic form) or selenised-yeast (organic form). As lactic acid bacteria (LAB) have been shown to incorporate Se as either organic or elemental (Nano-Se) there may be potential to use silage inoculant bacteria to improve the Se status of feed to provide the Se requirements of livestock. We screened twenty-seven LAB in MRS broth in the presence of sodium selenite for growth and uptake of Se as organic (selenocysteine and selenomethionine), inorganic (selenite and selenate) or/and Nano-Se, with the aim to identify potential candidates for a mini-silo study. Sodium selenite addition into the growth medium of LAB reduced growth rates but also resulted in the conversion of the inorganic sodium selenite into predominately Nano-Se and small quantities of organic-Se. Based on a rank analysis of growth and ability to take up (total Se content) and convert inorganic Se (Nano and organic Se content), three LAB were selected for further investigation as silage inoculants: *L. brevis* DSMZ (A), *L. plantarum* LF1 (B), and *L. plantarum* SSL MC15 (C). Each LAB was used as an inoculant within a grass mini-silo trial, either cultured in the presence of sodium selenite before inoculation or sodium selenite added to the inoculum at inoculation versus controls with no Se. The addition of sodium selenite either into the growth media of LAB or applied at inoculation of grass silage did not interfere with the ability of the LAB to act as a silage inoculant with no difference in silage fermentation characteristic between LAB with no Se added. The addition of sodium selenite either to the LAB growth medium or at inoculation resulted in the conversion of sodium selenite into Nano-Se and organic-Se (Nano-Se, ca. 10^3^ higher than organic), as previously shown in the screening trial. There was no difference between the three LAB for incorporation of Se or in silage quality, indicating the potential to develop silage inoculants to increase the bioavailable form of Se (elemental and organic) to livestock through conversion of inorganic forms during ensiling.

## Highlights

• Lactic acid bacteria convert sodium selenite into organic selenocysteine and elemental Nano-selenium.
• Lactic acid bacteria were selected on their ability to grow in a sodium selenite medium and to convert selenite to organic and Nano-Se.
• The ability of lactic acid bacteria to act as silage inoculants was not compromised by the addition of sodium selenite.
• Lactic acid bacteria inoculants can convert sodium selenite in silo to supplement livestock with organic and predominately Nano-Se.

## 1 Introduction

Selenium (Se) is an essential non-metallic trace element which is a constituent of more than twenty seleno-proteins that play critical roles in reproduction, thyroid hormone metabolism, DNA synthesis, and protection from oxidative damage and infection (Sunde, 2006). Typically, human diets, in the UK, have contained roughly 50% of the recommended daily allowance of Se (Rayman, 2000). Although Se deficiency symptoms are rare there are groups that are particularly susceptible, either due to low intake or poor up-take e.g. vegetarians and particularly vegans, people living in Se-deficient regions, people living with HIV or under-going kidney dialysis and the elderly. For these people and those with-out a well-balanced diet, there is an increasing reliance on dietary supplements to provide their essential minerals and vitamins including Se (Jackson et al., 2003). Brazil nuts (*Bertholletia excels*) are well known to be an excellent supplier of Se. However, depending on their origin a recent study from Brazil (Silva et al., 2017) showed that a single Brazil nut could provide between 11 (harvested from the Mato Grosso state) and 288% (from the Amazonas state) of the daily Se requirement for an adult man (70 μg/d) due to large variations in soil Se (<65.76 to 625.91 μg/kg) and soil conditions (acidity). As such, more reliable and accessible sources of Se are from animal products (fish, meat and milk) with the most common source in the typical Western diet coming from dairy and meat (Oster and Prellwitz, 1989). However, levels of organic Se in animal products are hampered by low animal up-take as forages world-wide frequently contain inadequate levels of essential minerals to meet the requirements of farmed livestock (Lee et al., 2018). The Se status in grazed crops is dependent on incorporation from soil. Soil types, which vary dramatically globally, influence Se concentrations where typically: laterite, yellow and red soils (developed under tropic or sub-tropic conditions) have relatively high Se (>0.3 ppm); chernozem, chestnut, calcic brown, desert and solonchak soils (developed under temperate steppe and desert conditions) have moderate Se (0.14–0.3 ppm) and brown earth, drab, dark brown, loessial soils (developed under the temperate humid/sub-humid conditions) are low in Se (<0.14 ppm; Tan et al., 2002). Furthermore, Se availability to plants depends on numerous soil factors including pH, redox, other mineral antagonism, organic matter content and fertilisation (Munier-Lamy et al., 2007).

Levels of Se supplementation for livestock range between 0.30–1.00 mg/kg DM, described as adequate, and 3.00–4.00 mg/kg DM, described as high (Puls, 1988). This wide supplementation range is associated with the different forms of Se supplements (organic vs inorganic), which range in bioavailability. An inadequate supply of Se to livestock can lead to reduced performance and production disorders such as retained placenta at calving and high somatic cell counts in milk, reducing milk quality and quantity and increasing the risk of mastitis (Weiss et al., 1990). Additionally, mineral deficiencies in newly born livestock can result in ill-thrift, brought about by a reduced ability to suckle, and white muscle disease, leading to stiffness, inability to stand and ultimately death (Lee et al., 2018). It is therefore common place to supplement ‘on-farm’ either through the incorporation of minerals into complete rations or by the provision of easily accessible mineral blocks (licks). Although supplementation with inorganic minerals, such as sodium selenite, should overcome these problems, it has been shown that livestock are less able to absorb inorganic Se, and ruminants to an even greater extent than monogastric livestock, with <50% absorbed (Wright and Bell, 1966). Furthermore, sodium selenite, the inorganic form of Se, is less efficiently converted into milk Se than its organic counterpart, Se-amino acids, giving rise to further deficiencies in the supply of Se to offspring and into product (Juniper et al., 2006).

Currently organic Se is predominately delivered as a selenised-yeast supplement, principally in the form of selenomethionine (Se-Met). Inorganic Se incorporation and conversion to organic forms has also been reported in algae (Skrivan et al., 2010; Travnicek et al., 2007) and certain bacteria including strains of lactic acid bacteria (LAB) converting sodium selenite into organic seleno-amino acids (Calomme et al., 1995), principally in the form selenocysteine (Se-Cys) and also full reduction to elemental Nano-Se (Bertolini et al., 2014; Eszenyi et al., 2011; Lamberti et al., 2011). The past 40 years have seen research and commercial development of a wide range of biological silage additives. The majority of these consist of lyophilized cultures of various strains of LAB. These products are re-hydrated and added to herbage at time of cutting in order that the micro-organisms they contain can dominate the epiphytic micro-flora and ‘direct’ the silage fermentation to optimise preservation and conserve the nutritional value of the ensiled crop. Traditionally selection of silage inoculants has been based upon two criteria, mainly: (a) rapid growth rates accompanied by the production of copious quantities of lactic acid, to depress herbage pH rapidly and decreased levels of ammonia and butyric acid in the resultant silage and to a lesser extent (b) an ability to inhibit the development of silage spoilage bacteria, yeasts and moulds and improve hygienic status and aerobic stability of the resultant silage (Spoelstra, 1991). Consideration has not been given to the possibility that the silage inoculant or its culture medium could be fortified with minerals essential for optimal livestock nutrition and the silage itself used as an effective delivery mechanism to counteract potential mineral deficiencies in ruminants and improve product quality (meat and milk). Here we screen a selection of LAB from two collection libraries for their ability to incorporate Se and convert to organic and Nano forms and subsequently their potential to act as a silage inoculant.

## 2 Materials and methods

The study consisted of two stages, firstly to screen a selection of LAB isolates for their ability to grow in the presence of sodium selenite and incorporate Se either in an organic, inorganic or elemental form; secondly to select the best performing isolates from the screen to carry out a grass mini silo study to determine their potential as silage inoculants to deliver Nano-Se or organic-Se when Se was added either to the growth medium or directly at ensiling.

### 2.1 LAB screening

#### 2.1.1 Isolates and viability

LAB isolates were supplied from the collections of the University of Bristol (Bristol Veterinary School, Somerset, UK) and Silage Solutions Ltd. (Aberystwyth, Ceredigion, UK) supplied as either a freeze-dried powder (21) or agar slopes (6). In total there were 12 species and 27 isolates (Table 1). Freeze-dried isolates were reconstituted in 0.5 ml of a sterile ¼ strength Ringers solution (Sigma-Aldrich Ltd, Gillingham, Dorset, UK) and an aliquot (100 μl) transferred to MRS broth (Oxoid Ltd, Basingstoke, Hampshire, UK) (10 ml) and grown overnight at 30 °C. A loop full of culture from the agar slopes were aseptically transferred to 10 ml of fresh MRS broth and grown overnight with the other isolates.

**Table 1:** Lactic acid bacteria species and isolates tested for viability.

Species	Isolates
*L. plantarum*	**LF1**, **L54**, CNCM I-3235, **MTD1**, **Lp3 A0905**, **MA16/4U**, **DSMZ 16627**, **SSL MC15**, JB012
*P. acidilactici*	**CNCM I-3237**, **MA18/5 M**, **NCIMB 30005**, SSL MC15
*L. casei*	JB005, **JB008**
*P. pentocaseous*	**NCIMB 12455**, **NCIMB 30044**
*L. salivarius*	**CNCM I-3238**, ASF361
*L. brevis*	**LB1 L1529**, **DSMZ 16680**
*L. buchneri*	Bio
*L. paracasei*	**NCIMB 30151**
*L. fermentum*	**NCIMB 30169**
*E. faecium*	M74
*L. acidophilus*	**ASF360**
*L. crispatus*	UoB

Isolates in **bold** were viable and used in the culture screen.

The standard growth conditions for LAB to reach the end-point is 24 h in broth cultures incubated at 30 °C (de Man et al., 1960). After 24 h, cell count was measured for each isolate to determine viability by pour plates. Briefly, a 1 ml aliquot was transferred to 9 ml of ¼ strength sterile Ringers with appropriate tenfold serial dilutions prepared. Viability was confirmed with cell counts around 2 × 10^9^ /ml of culture, of which 19 of the 27 were confirmed viable (Table 1).

#### 2.1.2 Cultures and identification of isolates for mini-silos

Each of the viable isolates was cultured in triplicate in 400 ml of either: MRS broth (Control, CMRS) or Se-enriched MRS broth (SeMRS) (315 mg sodium selenite added to 400 ml MRS). Cultures were inoculated with 0.1% v/v of overnight grown culture and incubated at 30 °C for 20 h. At the end of the incubation period each culture was subsampled to measure pH (10 ml) and enumerated, a 1 ml aliquot of the culture was aseptically transferred to a sterile petri dish to which molten MRS agar was poured and enumeration performed as with the viability test. The remaining sample was centrifuged at 2800 *g* for 20 min, the supernatant removed and the bacteria washed with saline and centrifuged for a further 20 min. A second saline wash was performed followed by a final wash in water to obtain a pure bacteria pellet. The weight of bacterial pellet was recorded and freeze-dried prior to Se determination (as described in 2.3).

### 2.2 Mini silo trial

#### 2.2.1 Treatments and inoculum

There were 3 treatments for each of the three isolates identified in the culture screening (A, B, C) each set up in triplicate for each opening time of the mini silos (1, 2, 8, 30 and 90 days) in addition to a negative control with no inoculum. This gave a total of 150 mini silos: Isolate LAB (A, B or C) with no Se enrichment (LAB; n = 45); Isolate LAB (A, B or C) grown in Se enriched media (LABSe; n = 45); Isolate LAB (A, B or C) with Se added at ensiling (LAB + Se; n = 45) and a control with no inoculant (CON n = 15).

The LAB culture medium contained 4 × 10^12^ CFU/l of MRS broth and the inoculum was added to the wilted grass at an application of LAB 1 × 10^6^ per g fresh weight (FW). The relevant Se-inoculum contained 0.5 g Se for 1 t silage FW to equate to ca. 2.5 mg/kg DM based on mean silage DM. Ruminant Livestock requirements equate to circa 0.3 mg/kg DM of the total diet, so the aim was to produce a silage which could then form part of a Total Mixed Ration to deliver the required level of Se. This was either added to the growth medium as sodium selenite prior to culturing (LABSe) or at ensiling (LAB + Se).

Permanent pasture of ca. 0.5 tonnes FW was cut with a plot harvester (Haldrup 1500, J. Haldrup, Logstor, Denmark) and laid-out on top of new unused silage sheet to wilt for 24 h. Although a specific DM target was not set, the aim was to simulate a low DM silage typical of UK conditions, to test for the viability of the Se containing inoculants. The grass was then passed through a forage harvester. Kilner® jars (1 l, Lakeland Ltd., Windermere, Cumbria, UK) were used as mini silos and pre-weighed before filled with ca. 1 kg FW and sealed, with the accurate weight recorded for each mini silo.

#### 2.2.2 Mini silo opening, sampling and analysis

At day 1, 2, 8, 30 and 90 the relevant jars (n = 30) were opened. On the day of opening water extract of each silage was produced by mixing 50 g FW with 250 ml of deionized water, in a stomacher for 2 min. A glass electrode of a Jenway 3320 calibrated pH meter, was used to measure the pH of the homogenized water extract (Jenway, Stone, Staffordshire, UK). Fresh samples of silage (ca. 200 g FW) were frozen and sent on dry-ice for assessment of fermentation parameters (Ammonia-N, Volatile Fatty Acids (VFA) and Lactic acid). A water extract was prepared using 10 g FW and 90 ml of distilled water, stored at 4 °C for 16 h before being filtered and the filtrate used for subsequent analysis. This was used to determine the analytes by a HPLC method as described by Canale et al. (1984). The remaining silage was freeze-dried and ground for total Se assessment, speciation was performed only on the day 90 opening sample (as described in section 2.3).

### 2.3 Selenium analysis

#### 2.3.1 Total selenium

An aliquot of LAB or silage sample (0.1 g DM) was accurately weighed into a 40-ml ultra-clean glass digestion vial along with 3 ml concentrated nitric acid. The digestion vials were heated at 120 °C for 1 h on a hot block before 1 ml of hydrogen peroxide was added and heated for a further hour. The vials were loosely capped to ensure the reflux of the acid during digestion. After the 2 -h digestion period, digests were left to cool in a fume cupboard before dilution to the mark with deionised water. An aliquot of the sample digest (0.2 ml) was transferred into a clean 1 ml auto-sampler vessel and diluted to the mark with deionised water. The samples were then analysed by Ultra-Violet Hydrogen-Generation Atomic Fluorescence Spectrometry (UV-HG-AFS) using the Millennium Excalibur System (PS Analytical, Orpington, Kent, UK). The system used 0.7% m/v sodium hydrogen bromide in 0.1 M sodium hydroxide as the reductant and 10% v/v hydrochloric acids as the reagent blank. A pre-reductant solution (50% v/v hydrochloric acid with 5% m/v potassium bromide) was used to reduce selenate (SeVI) to selenite (SeIV) online. Calibration standards of 0–20 ng/ml were prepared in reagent blanks.

#### 2.3.2 Selenium speciation analysis

A one-step enzymatic extraction with protease XIV (Sigma-Aldrich Ltd., Gillingham, Dorset, UK) was applied for selenium speciation. An aliquot of LAB or silage sample (0.1 g DM) was accurately weighed into a 15-ml ultra clean polypropylene centrifuge tube followed by the addition of 20 mg enzyme and 8 ml phosphate buffer (60 mM, pH 7.4). The samples were then capped tightly and put on an automatic shaker for 24 -hs at room temperature. After proteolysis, the samples were centrifuged for 20 min. The supernatants were filtered by 0.45 μm PTFE syringe filters (Sigma-Aldrich Ltd.) and transferred into a clean vial. These solutions were then further diluted by deionised water and analysed by HPLC-UV-HG-AFS using the Millennium Excalibur System (PS Analytical). All Se which was not identified as inorganic (SeVI or SeIV) or organic (seleno-amino acids) was presumed to be Nano-Se as previously proposed by Eszenyi et al. (2011) and Xia et al. (2007). This does over estimate total Nano-Se slightly, but as previously reported when sodium selenite is supplied at greater than 64 mg/l Nano-Se accounts for over 90% of total Se within LAB cultures.

### 2.4 Statistical analysis

For the lab screening trial, the ability of each culture to sequester Se and convert to either Nano-Se or organic-Se were performed using a student’s *t*-test (CMRS vs. SeMRS) with the confidence interval at P < 0.05. Mean data for the CMRS and SeMRS was then plotted and analysed by concordance correlation for Se-content and percentage conversion (organic and Nano). Finally, a correlation analysis was conducted between Se-enrichment and CFU Log_10_/ml to select the three best performing isolates (A, B and C) in terms of the key criteria: total Se uptake, percentage conversion and bacterial growth.

A general ANOVA was performed on the time course for changes in pH, VFA, lactic acid and Ammonia-N for all treatments with Days at Opening (1, 2, 8, 30 and 90 days) * Treatment (LAB + LABSe + LAB + Se) as the treatment structure. On day 90 samples pairwise comparisons were performed as a general ANOVA with Con vs Treatment (LAB + LABSe + LAB + Se); Inoculum (A vs B vs C); LAB application (LAB vs LABSe vs LAB + Se) and interaction (Inoculum * LAB application). All statistical operations were performed using GenStat 64-bit Release 17.1 (PC/Windows 7) 11 Copyright 2014, VSN International Ltd.

## 3 Results

### 3.1 LAB screening

Of the 27 LAB isolates across 12 species received, 19 were deemed viable across 9 species based on CFU counts from an initial culture (Table 1). Growth of the LAB in control MRS broth (CMRS) and MRS broth containing sodium selenite (SeMRS) are reported in Table 2 along with culture pH. CMRS growth ranged from 9.6 to 10.9 Log_10_ CFU/ml whereas for SeMRS the range was 6.6–9.6 Log_10_ CFU/ml, with growth of all LAB reduced as a response to the addition of sodium selenite. pH of the cultures was increased with the addition of sodium selenite with CMRS ranging from 3.77 to 4.39 with a mean of 3.96 and SeMRS ranging from 4.43 to 5.81 with a mean of 4.76.

**Table 2:** Growth of isolates and culture pH with and without selenium selenite (means ± standard deviation).

Bacteria	CFU Log^10^/mL (CMRS)	CFU Log^10^/mL (SeMRS)	pH (CRMS)	pH (SeMRS)
*L. plantarum* L54	10.2 (±0.03)	9.3 (±0.02)	3.77 (±0.167)	4.70 (±0.154)
*L. plantarum* LF1	9.7 (±0.01)	9.6 (±0.03)	3.86 (±0.132)	4.43 (±0.123)
*L. plantarum* MTD1	9.7 (±0.02)	8.5 (±0.03)	3.77 (±0.164)	4.73 (±0.165)
*P. acidilactici* CNCM I-3237	9.8 (±0.02)	8.4 (±0.01)	4.36 (±0.195)	5.82 (±0.143)
*L. salivarius* CNCM I-3238	9.6 (±0.03)	7.6 (±0.02)	3.84 (±0.150)	4.66 (±0.174)
*L. brevis* LB1 L1529	10.1 (±0.02)	7.7 (±0.03)	3.79 (±0.176)	4.53 (±0.114)
*L. planatrum* Lp3 A0905	10.0 (±0.04)	7.7 (±0.01)	3.83 (±0.173)	4.73 (±0.174)
*L. planatrum* MA16/4U	10.2 (±0.02)	9.2(±0.03)	4.10 (±0.175)	4.43 (±0.154)
*P. acidilactici* MA18/5 M	10.9 (±0.03)	9.1 (±0.02)	4.10 (±0.187)	4.52 (±0.135)
*P. pentocaseous* NCIMB 12455	10.1 (±0.01)	8.7 (±0.03)	3.94 (±0.164)	4.54 (±0.163)
*L. plantarum* DSMZ 16627,	9.9 (±0.05)	7.4 (±0.02)	3.74 (±0.188)	4.58 (±0.154)
*L. paracasei* NCIMB 30151,	9.9 (±0.02)	7.4 (±0.01)	3.74 (±0.145)	4.63 (±0.137)
*P. acidilactici* NCIMB 30005,	9.7 (±0.02)	7.8 (±0.02)	4.72 (±0.136)	4.45 (±0.164)
*P. pentocaseous* NCIMB 30044.	9.6 (±0.04)	6.6 (±0.05)	3.79 (±0.179)	5.67 (±0.157)
*L. brevis* DSMZ 16680,	10.6 (±0.03)	8.2 (±0.02)	3.79 (±0.178)	4.61 (±0.138)
*L. fermentum* NCIMB 30169	10.5(±0.03)	8.4 (±0.03)	4.39 (±0.187)	4.47 (±0.156)
*L. plantarum* SSL MC15	10.5 (±0.01)	8.1 (±0.01)	3.79 (±0.155)	4.70 (±0.139)
*L. casei* JB008	10.3 (±0.02)	8.3 (±0.03)	4.08 (±0.138)	5.81 (±0.247)
*L. acidophilus* ASF 360	10.5 (±0.03)	8.7 (±0.02)	4.14 (±0.156)	4.50 (±0.157)

CFU, Colony Forming Units; CMRS, Control Medium; SeMRS, Sodium selenite containing medium.

**Fig. 1:**
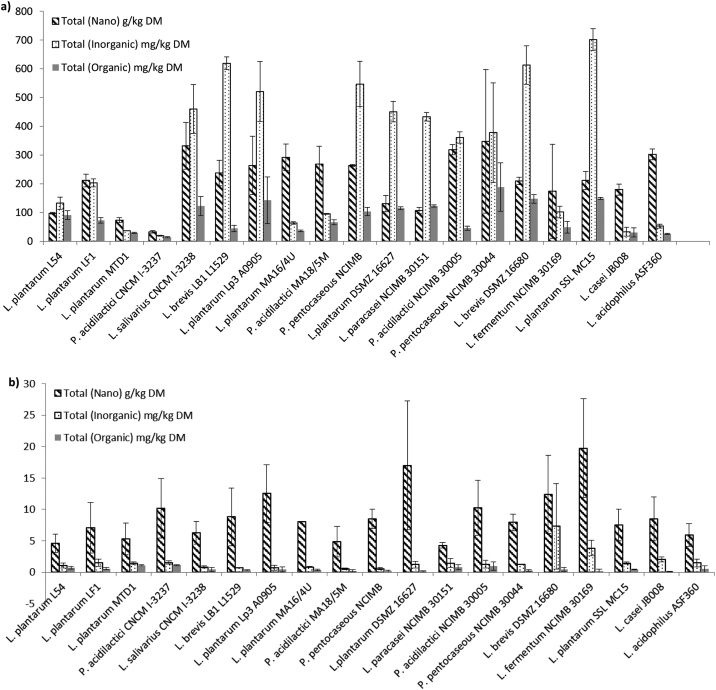
Se content of the isolates cultured in MRS broth with sodium selenite (SeMRS; a) and without (MRS; b). Means presented with standard error of the mean.

**Fig. 2:**
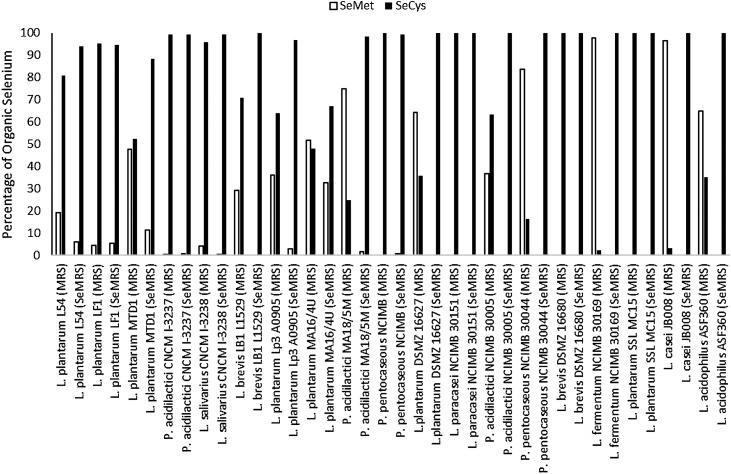
Percentage of total organic selenium as Selenomethionine and Selenocysteine of isolates cultured in MRS broth with sodium selenite (SeMRS) and without (MRS).

There was a wide range of ability to incorporate Se by the LAB both between and within isolates, with the majority of total Se being neither inorganic nor organic and therefore deemed to be insoluble elemental or Nano-Se (Fig. 1a). When the isolates were cultured in CMRS broth Total Se (Nano, Inorganic and Organic) ranged from 4.23 to 19.7 g/kg DM, with inorganic and organic ranging from 0.55 to 7.31 and 0.12–1.14 mg/kg DM, respectively (Fig. 1b). The same isolates cultured in SeMRS showed ranges for Total Se (Nano, Inorganic and Organic) from 33.0 to 347 g/kg DM, with inorganic and organic from 19.9 to 701 and 14.5–189 mg/kg DM, respectively (Fig. 1a). The percentages of the organic Se within the isolates as SeMet and SeCys when either cultured in CMRS or SeMRS are shown in Fig. 2. Within CMRS SeMet ranged from 0 to 97.7% with an average of 37.5%, and SeCys ranged from 2.31 to 100% with an average of 62.0%. For SeMRS, SeMet ranged from 0 to 32.8% with an average of 3.28%, and SeCys ranged from 67.2 to 100% with an average of 97.7%, where all isolates increased the proportion of SeCys in the presence of sodium selenite.

Based on the rank performance of all isolates for: i. growth (with a cut-off 8.0 CFU Log^10^/ml in SeMRS), ii) total Se uptake and ii) conversion to organic Se, two isolates were chosen. *L. brevis* DSMZ was assigned as isolate A and *L. plantarum* SSL MC15 assigned as isolate C. A third isolate L. *plantarum* LF1 was assigned as isolate B, which was selected on its high growth rate and its low variation in Se uptake and conversion, rather than total uptake and conversion per se (Table 2 and Fig. 1a).

### 3.2 Mini silos

**Fig. 3:**
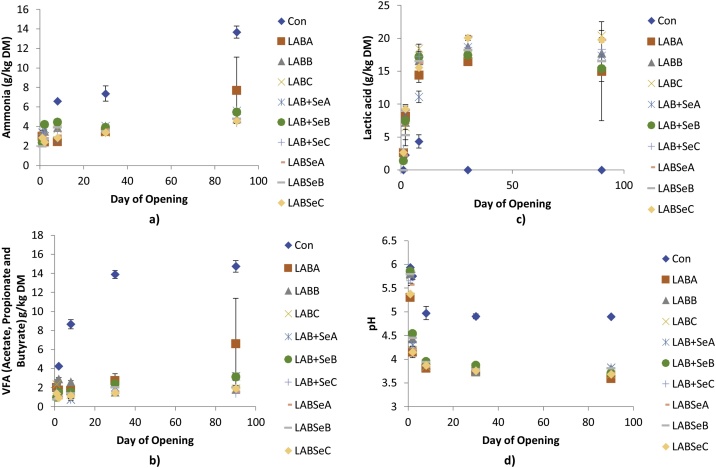
Mini silo silage quality parameters at opening. a) Ammonia-N; b) VFA, Volatile Fatty Acids’ c) Lactic Acid; d) pH. Con, Control with no inoculant; LAB A, Inoculant A ensiled; LAB B, Inoculant B ensiled; LAB C, Inoculant C ensiled; LAB + Se A, Se added to inoculant A at ensiling; LAB + Se B, Se added to inoculant B at ensiling; LAB + Se C, Se added to inoculant C at ensiling; LABSe A, Se cultured with inoculant A pre-ensiling; LABSe B, Se cultured with inoculant B pre-ensiling; LABSe C, Se cultured with inoculant C pre-ensiling. Means presented with standard error of the mean. Significance between treatments at P < 0.05 represented here by lack of overlapping error bars.

Temporal parameters of silage quality are presented in Fig. 3 across the five opening periods of the trial. Ammonia-N showed temporal increases from day 0 to day 90 for all silages but with significantly higher increases (P < 0.05) for CON than all LAB treatments from day 8 onwards. LABA had a high variance at day 90 due to one replicate showing higher levels of ammonia-N. Except for CON, lactic acid temporally increased to day 30 for all LAB treatments. At day 90 variance was higher especially in relation to LABA, due to one replicate as already indicated, but no further increase in lactic acid was observed between day 30 and 90 for all treatments. For CON, lactic acid increased to day 8 and was significantly lower than LAB + SeA which in turn was lower than all other treatments, which were comparable to each other. At days 30 and 90 no lactic acid was detected in any of the CON replicates. For total VFA there was little temporal increase from day 1 to day 90 for all treatments with the exceptions of LAB + SeA, LAB + SeB, LABA and CON. For both LAB + SeA and LAB + SeB there was numerically higher VFA concentrations at day 90 than the other treatments except for LABA, which had a higher mean and large variance as already discussed for lactic acid and ammonia-N. Total VFA for CON showed an increase over time to day 30 before plateauing, with all openings from day 2 higher than the other treatments. pH declined rapidly over the first 2 days for all treatments, except for CON, reaching plateau by day 5. For CON, pH saw no decline from day 1 to day 2 followed by a less extreme decline to day 5 before reaching plateau at a higher pH than the other treatments.

The full silage quality and Se status of the final day 90 opening is reported in Table 3 with pairwise comparisons of the main treatments and interactions. For all silage quality parameters there was a difference between CON, with no inoculant, and all other treatments that used either LAB A, B or C in the presence or absence of sodium selenite (LAB, LABSe or LAB + Se) with no difference between inoculum treatments. CON compared to all other LAB based treatments had lower DM and lactic acid and higher pH, acetate, propionate, butyrate, ethanol, total VFA and ammonia-N.

**Table 3:** Mini silos fermentation characteristics and selenium status at day 90.

		LAB			LABSe			LAB + Se				P			
	Con	A	B	C	A	B	C	A	B	C	S.e.d.	CvT	Inoc.	LAB	Int.
**Silage Quality**															
Dry matter (g/kg)	13.8	16.6	16.6	16.3	16.5	15.9	16.4	16.1	16.0	16.3	0.215	<0.001	0.332	0.546	0.475
pH	4.90	3.60	3.80	3.69	3.71	3.81	3.69	3.83	3.71	3.83	0.067	<0.001	0.342	0.200	0.100
Acetate (g/kg DM)	3.33	2.20	1.15	1.35	1.31	1.04	1.40	1.36	1.04	1.40	0.386	<0.001	0.143	0.360	0.396
Propionate (g/kg DM)	2.41	1.01	0.18	0.07	0.02	0.06	0.02	0.18	0.17	0.000	0.417	<0.001	0.341	0.291	0.507
Butyrate (g/kg DM)	8.59	3.22	0.53	0.48	0.25	0.90	0.44	1.54	1.72	0.07	1.487	<0.001	0.348	0.617	0.516
Ethanol (g/kg DM)	4.02	2.33	1.88	1.72	1.58	1.99	1.67	1.75	2.21	1.72	0.513	0.005	0.540	0.721	0.674
Total VFA (g/kg DM)	14.7	6.60	1.86	1.90	1.59	2.02	1.90	3.19	3.14	1.38	2.280	<0.001	0.328	0.513	0.481
Lactic acid (g/kg DM)	0.000	15.1	17.8	20.4	19.8	16.6	19.8	17.4	15.4	18.4	3.73	0.001	0.429	0.761	0.809
NH_3_-N (g/kg DM)	13.7	7.70	4.77	4.93	4.50	4.80	4.57	5.63	5.47	4.37	1.627	<0.001	0.398	0.495	0.641
**Selenium (mg/kg DM)**															
SeMet	0.05	0.07	0.03	0.04	0.05	0.04	0.06	0.04	0.05	0.06	0.012	0.153	0.048	0.975	0.065
SeCys	0.002	0.010	0.002	0.001	0.038	0.047	0.058	0.094	0.036	0.077	0.0108	<0.001	0.018	<0.001	0.005
Se IV	0.03	0.14	0.01	0.15	0.06	0.01	0.03	0.07	0.03	0.06	0.029	<0.001	0.001	0.003	0.091
Se VI	0.000	0.010	0.000	0.019	0.010	0.017	0.009	0.023	0.017	0.011	0.0099	0.366	0.870	0.463	0.348
Total Se (Nano)	0.20	0.18	0.14	0.17	2.76	2.84	2.09	2.73	2.86	2.83	0.779	<0.001	0.475	<0.001	0.557

Con, Control; A, Inoculant A; B, Inoculant B; C, Inoculant C; LAB, Inoculant with no Se; LABSe, Inoculant cultured with sodium selenite; LAB + Se, Inoculant with sodium selenite added at ensiling; VFA, Volatile Fatty Acids; NH_3_-N, Ammonia Nitrogen; SeMet, Selenomethionine; SeCys, Selenocysteine; Se IV, selenite; Se VI, selenate. S.e.d., Standard error of the difference; CvT, Control versus Treatments; Inoc, A versus B versus C; LAB, LAB versus LABSe versus LAB + Se; Int., Interaction.

SeMet was not different between CON and inoculated treatments, nor between LAB treatments (LAB, LABSe or LAB + Se) with no interaction with inoculum used, although LAB B was lower in SeMet than either LAB A or LAB C. For SeCys, CON was lower than all treatments with LABSe and LAB + Se higher than LAB. LAB B was lower than LAB A and LAB C but only within LAB + Se, hence a significant interaction effect. Inorganic Se IV (selenite) was lower for CON than all treatments but unlike SeCys, LABSe and LAB + Se were lower than LAB. LAB B was also lower than LAB A and LAB C with no interaction effect. Inorganic Se VI (selenate) was not different across all treatments including CON. Total Se (including Nano-Se) was lower for CON than all treatments and lower for LAB than LABSe and LAB + Se, with no difference between inoculants used (A, B or C).

## 4 Discussion

### 4.1 LAB screening and Se uptake and conversion

Sodium selenite addition into the growth medium of LAB reduced growth rates but also resulted in the conversion of the inorganic sodium selenite into predominately Nano-Se as previously reported for *M. bovis* (Biswas et al., 2011), *P. fluorescens, B. subtilis* (Garbisu et al., 1996), *S. maltophilia* (Bertolini et al., 2014) and the LAB *L. reuteri* Lb2 BM (Lamberti et al., 2011), *L. casei, S. thermophilus, B. bifidum, L. acidophilus* LA-5, and *L. helveticus* (Eszenyi et al., 2011). Formation of Se-Cys from sodium selenite was also noted within *L. reuteri* LB2 BM by Lamberti et al. (2011) who also reported the reduced growth rates in the presence of Se. Se-exposure has been shown to result in the over-expression of two heat shock proteins (GroEL and GrpE) which have both been implicated in environmental stress responses (Desmond et al., 2004). This stress response was confirmed within the current screen where all LAB isolates reduced growth rates in the presence of sodium selenite. Xia et al. (2007) reported an increase in bacteria growth for *L. bulgaricus* at a low dose of 1 mg sodium selenite/l with growth unaffected between 4 and 16 mg/l and a reduction observed with application >32 mg/l. In the current study sodium selenite was applied to the medium at 787.5 mg/l with LAB isolates showing a significant reduction in growth.

Calomme et al. (1995) reported that LAB incorporated Se intracellularly within its proteins as both SeMet and SeCys, with SeCys the predominant seleno-amino acid. The seleno-proteins in LAB include at least three forms of formate dehydrogenase, glycine reductase A and B and NiFeSe hydrogenase with all these enzymes including SeCys at the active site (Lamberti et al., 2011). In contrast yeast and plants predominately store and assimilate Se as SeMet (de Souza et al., 2000; Guo and Wu, 1998). In our screening most LAB in the absence of sodium selenite showed levels of both SeCys and SeMet with SeCys predominate but not in all cases, where 7 of the 19 contained higher levels of SeMet. However, when grown in the presence of excess Se through the addition of sodium selenite, all LAB showed a greater proportion of SeCys at the expense of SeMet as reported by Calomme et al. (1995). The formation of Nano-Se by LAB has been reported as a detoxification process reducing Se (IV) to non-soluble Se (0) to be stored as an electron-dense and amorphous granule deposited in the cytoplasm and extracellular space (Garbisu et al., 1996; Xia et al., 2007; Eszenyi et al., 2011). Xia et al. (2007) reported that accumulation of Nano-Se commenced at concentrations above 4 mg sodium selenite/l culture medium, with concentration increasing as sodium selenite increased within the medium to the highest dose of 64 mg/l where 90% of the Se within the LAB was Nano-Se (11.2 g/kg DM). In the current study Nano-Se (predicted as non-organic/inorganic insoluble Se) ranged between 32.0–332 g/kg DM across the LAB cultures with a substantially higher level of sodium selenite in the culture medium than Xia et al. (2007) of 787.5 mg/l. This range in capability of LAB to convert and store Nano-Se has also been reported previously by Eszenyi et al. (2011), with variance across 5 LAB strains of 10–35 g Nano-Se/kg DM when the culture medium contained 200 mg sodium hydrogen selenite/l. The current study therefore confirms that selected LAB, used for silage inoculation, will utilise sodium selenite to form Se-Cys up to biological limits and then preferentially convert to Nano-Se as means of detoxification.

### 4.2 Ability of Se incorporating LAB to act as silage inoculants

The first aim of the current study was to select three LAB cultures based on the rank performance of all isolates for: i) growth, with a cut-off 8.0 CFU Log_10_/ml in SeMRS, as it was determined that growth rate was critical for a good silage inoculant, ii) total Se uptake and, iii) conversion to organic Se, as this was important for Se bioavailability. From this rank assessment two isolates were chosen: *L. brevis* DSMZ assigned as isolate A and *L. plantarum* SSL MC15 assigned as isolate C. However, as the second part of the study was to assess the potential as a silage inoculant the decision was taken to choose the third isolate based on growth rate in SeMRS and so the third isolate *L. plantarum* LF1 assigned as isolate B was selected on its ability to grow in SeMRS (Table 2) rather than the total rank assessment.

The two *L. plantarum* strains selected are facultatively homofermentative species producing predominantly lactic acid as an end-product of hexose fermentation (Merry et al., 1995; Weinberg and Muck, 1996), the strain of *L. brevis* is obligately heterofermentative producing a mixture of lactic, acetic and ethanol from hexose fermentation. Effective strain selection has been an important part of inoculant development with typical measures of silage quality such as lactic acid production, speed of fermentation and protein quality as key determinants (Davies et al., 1998). The fermentation profiles (Fig. 3) for all inoculant treatments, other than the CON with no inoculant, showed a typical fermentation pattern with a rapid drop in pH driven by high production of lactic acid, low production of VFA and high protection of forage protein (as indicated in the low ammonia-levels) (Merry et al., 1995; Davies et al., 1998). The slight discrepancy with the LABA treatment is more likely related to a poor seal reducing anaerobicity with a degree of secondary fermentation, VFA formation and greater protein breakdown (ammonia-N), but could also be due to the fact this treatment used a heterofermentative inoculant which is known for reducing the speed of acidification in the silo due to the production of the weaker acetic acid as part of its metabolism (*L. brevis* DSMZ). However, the same could not be said for the CON treatment which shows a poor fermentation stability and a failure of fermentation with high pH, low levels of lactic acid production and high levels of VFA indicative of a poorer and potentially clostridial fermentation, and nutritional value (Davies et al., 1998). Although, not a direct aim of the project, the lack of a stable fermentation in the control mini silo treatment without inoculant, showed the potential of each inoculant either in the presence or absence of Se to improve the quality of the fermentation and nutritional value of the crop. There was no difference between inoculant used (A vs. B vs. C) in fermentation parameters or whether Se was present or not (LAB vs. LABSe + LAB + Se), or when Se was added (LABSe vs LAB + Se).

Final silage quality parameters (Day 90; Table 3) confirmed the temporal fermentation patterns where CON without inoculant was significantly poorer than all LAB silage treatments with lower dry matter (indicative of silage losses of water-soluble carbohydrate and protein) and lactic acid; and higher pH, VFAs (acetate, propionate and butyrate; indicative of a greater influence of either Enterobacteria, clostridial or/and potentially less effective lactic acid bacterial fermentation), ethanol, and ammonia-N (also indicative of loss of protein). For silage quality parameters at Day 90 there was no difference between inoculants used (A vs. B vs. C) or whether Se was present or not (LAB vs. LABSe + LAB + Se), or when Se was added (LABSe vs LAB + Se) with all performing significantly better than the control. Seppala et al. (2014) investigated the potential of adding Se to silage as sodium selenate at 10, 50 or 500 mg / kg, either directly in water or with an acid-based additive (formic, propionic, ammonium formate and benzoic acid; 6 g/kg DM). They also reported no effect of Se compared to the control on silage quality parameters when just added to the forage in water prior to ensiling. Although, they did report reduced lactic acid production at the highest inclusion level of sodium selenate (500 mg/kg) within the acid-based additive treatments.

SeMet in the silages was not different between the control (with no inoculant) and LAB treated silage, or when Se was applied at the culturing (LABSe) or ensiling stage (LAB + Se). However, inoculant strain was significantly different with B lower than A and C. This would suggest that the majority of the SeMet is from the crop itself with a small contribution from the silage microbiota. Most plant organic Se is in the form of SeMet (Guo and Wu, 1998), hence the proportionally higher SeMet content compared to SeCys in the treatments which did not contain added Se (CON and LABA, LABB and LABC). The lower levels within isolate B silages may have been due to the lower incorporation ability of *L. plantarum* LF1, than the other isolates. LAB B was selected on growth potential, which may have influenced the subsequent silage microbiota and so reduced SeMet compared to the other silages where LABs A and C have been shown to incorporate more Se. Results of the earlier screening study and that of previous research (Calomme et al., 1995; Lamberti et al., 2011) for the ability of LAB to convert sodium selenite into SeCys were confirmed in the Day 90 silage samples with all LABSe and LAB + Se silages showing elevated levels over CON and LAB treatments where no sodium selenite was added. Similar to SeMet LAB B was lower than LAB A and C but only when Se was added at the ensiling stage (LAB + Se), which may have been due to lower Se uptake pressure when provided outside the culturing medium.

Sodium selenite levels were lower for CON than the LAB treated silages and as previously indicated for LAB B than either LAB A or C. However, this difference was more pronounced for the LAB without additional Se. This suggests the result is more associated with the LAB cultures themselves and their low basal level of Se (IV), rather than the effect of the additional Se and its conversion, as the results mirror the patterns found within Fig. 1b of the screen study when cultured in MRS without sodium selenite. The low levels of sodium selenite in all silages, either in treatments where it has been added is indicative of the reported ability of LAB to convert sodium selenite, predominantly into Nano-Se (Garbisu et al., 1996; Xia et al., 2007; Eszenyi et al., 2011). Sodium selenate was not different across all silages and found in low levels (tr – 0.023 mg/kg DM). Previously when sodium selenate was added to forage, either in water or as part of an acid-based additive during ensiling, the level of sodium selenate found in the resultant silage was comparable to the level initially added (Seppala et al., 2014) suggesting that LAB are unable to convert sodium selenate into either organic or Nano-Se.

Most of the sodium selenite added to the silages either within the culture medium or at ensiling was converted to insoluble Nano-Se (as not detected as inorganic or organic forms and evidenced by the red particulate pellet formed) with no difference between LAB strain. This would suggest the potential to use silage inoculants to convert potentially toxic sodium selenite into Nano-Se to supplement livestock. However, the potential of Nano-Se for ruminants has yet to be fully elucidated as Nano-Se has been reported to be inert and so bioavailability may be reduced (Garbisu et al., 1996). Hudman and Glen (1985) reported the formation of elemental Nano-Se in the rumen by *B. ruminicola’s* reduction of selenite, although no assessment of availability was determined. Whereas, Wang et al. (2007) in mice found that Nano-Se was a highly effective antioxidant, without high toxicity properties, compared with sodium selenite. It also had the same effect of activating glutathione peroxidase and thioredoxin reductase enzymes as seleno-amino acids. Preliminary trials with sheep from this group have shown Nano-Se to have high availability and incorporation of Se into plasma, muscle and wool (Lee et al., 2016) with low levels of toxicity compared with inorganic and organic Se (Lee et al., 2017). However, more research is required to fully validate this approach in livestock feed, especially concerning bioavailability and thus determine the appropriate concentration of Nano-Se to be included within the silage.

## 5 Conclusions

Sodium selenite addition into the growth medium of LAB reduced growth rates but also resulted in the conversion of the inorganic sodium selenite into predominately Nano-Se. Based on a rank analysis of growth and ability to take up (total Se content) and convert inorganic Se (Nano and organic Se content), three LAB: *L. brevis* DSMZ, *L. plantarum* LF1, and *L. plantarum* SSL MC15 were selected for a mini-silo trial. The addition of sodium selenite either into the growth media or applied at inoculation of grass silage did not interfere with the ability of the LAB to act as a silage inoculant with no difference shown in silage fermentation characteristic between LAB with no Se added. All selected LABs as silage inoculants showed the ability to convert sodium selenite into Nano and Organic-Se (Nano ca. 10^3^ higher than organic). There is potential to develop silage inoculants to increase the bioavailable form of Se (Nano and organic) to livestock through conversion of inorganic forms during ensiling.

## Conflict of interest

There is no conflict of interest for any of the authors.
